# Postoperative Respiratory Impairment Is a Real Risk for Our Patients: The Intensivist's Perspective

**DOI:** 10.1155/2018/3215923

**Published:** 2018-04-03

**Authors:** Vidya K. Rao, Ashish K. Khanna

**Affiliations:** ^1^Divisions of Cardiothoracic Anesthesiology and Critical Care Medicine, Department of Anesthesiology, Perioperative and Pain Medicine, Stanford University School of Medicine, Stanford, CA 94305, USA; ^2^Center for Critical Care, Department of Outcomes Research, Anesthesiology Institute, Cleveland Clinic, Cleveland, OH 44195, USA

## Abstract

Postoperative respiratory impairment occurs as a result of a combination of patient, surgical, and management factors and contributes to both surgical and anesthetic risk. This complication is challenging to predict and has been associated with an increase in mortality and hospital length of stay. There is mounting evidence to suggest that patients remain vulnerable to respiratory impairment well into the postoperative period, with the vast majority of adverse events occurring during the first 24 hours following discharge from anesthesia care. At present, preoperative risk stratification scores may be able to identify patients who are particularly prone to respiratory complications but cannot consistently and globally predict risk in an ongoing fashion as they do not incorporate the impact of intra- and postoperative events. Current postoperative monitoring strategies are not always continuous or comprehensive and do not dependably identify all cases of respiratory impairment or mitigate their sequelae, which may be severe and require the use of increasingly limited intensive care unit resources. As a result, postoperative respiratory impairment has the potential to cause significant downstream effects that can increase cost and adversely impact the care of other patients.

## 1. Introduction

Respiratory impairment in the postoperative period is a significant contributor to morbidity and mortality following surgery and anesthesia [1]. In 2015, the Agency for Healthcare Research and Quality rated postoperative respiratory failure as the 4th most common patient safety event, with other studies indicating an associated increase in mortality and hospital length of stay [2]. There are significant data to suggest that the risk for respiratory compromise exists well beyond the duration of care in the postanesthesia care unit (PACU) and may be the highest in the first 4 to 6 hours following PACU discharge [3–6]. Up to 88% of respiratory events occur in the first 24 hours following the completion of anesthesia, and many of these cases appear to be preventable [3, 6]. While postoperative respiratory impairment can have tragic consequences for afflicted patients, there may be widespread downstream effects, particularly when critical care resources or unplanned intensive care unit (ICU) admission is required. The use of limited critical care resources can be financially burdensome, adversely impact the delivery of appropriate care to other patients, and affect the workflow and well-being of clinicians in critical care settings [7]. The purpose of this review is to describe the problem of postoperative respiratory impairment, highlight its impact on the intensive care unit, and emphasize the role of anesthesiologists in mitigating this complication.

## 2. Scope of the Problem

Postoperative pulmonary complications occur as a result of the interplay between modifiable and nonmodifiable comorbid conditions, surgical factors, persistence of intraoperative derangements in respiratory physiology, residual anesthetic effects, and the use of opioids and other sedating medications in the perioperative period. Despite the magnitude of this concern and the potential for catastrophic consequences in affected patients, no universal definition has been established. The published literature on this topic demonstrates significant variability in the characterization and identification of this complication, including hypoxemia, hypercarbia, hypoventilation, naloxone administration, or as part of a composite metric of postoperative pulmonary complications which include a wide variety of pathologies. Furthermore, the timing of what is classified as postoperative respiratory impairment varies considerably, ranging from the time spent in the postanesthesia care unit (PACU) to multiple days following surgery. The reported incidence ranges from 0.3 to 17% depending upon the metric evaluated [1, 8–10]. Institutional variability in anesthetic practice and postoperative monitoring strategies further precludes aggregation and generalizability of data. Thus, there is difficulty in accurately assessing the true incidence of postoperative respiratory impairment.

## 3. Mechanisms of Postoperative Respiratory Impairment

Respiratory physiology is dramatically altered with the initiation of anesthesia, particularly general anesthesia [11]. There is notable depression of central respiratory drive, and even low anesthetic levels can diminish compensatory responses to hypoxia and hypercarbia. Respiratory muscle tone is also altered, precipitating anatomic airway obstruction [12], which can persist even with low levels of residual neuromuscular blockade. Functional residual capacity is also diminished [13], the diaphragm displaced, and many patients develop pulmonary atelectasis, which has been visualized on computed tomography studies in anesthetized patients [14].

These intraoperative changes persist to varying degrees into the postoperative period, often many hours beyond the time spent in the PACU. Ventilatory response to hypoxia and hypercarbia can remain blunted, particularly while patients are being treated with opioids for acute pain. Atelectasis has been identified on CT scan 24 hours postoperatively with concomitant impairment of effort-dependent pulmonary function tests [15]. Functional residual capacity reaches its nadir at 1 to 2 days postoperatively and can remain diminished for upto 5 to 7 days afterwards. One study suggested that normalization of respiratory physiology could take up to 6 weeks [11, 16].

## 4. Predictors of Postoperative Respiratory Impairment

In recent years, significant effort has been devoted to risk stratification and identification of patients with increased vulnerability for developing respiratory impairment. Multiple surgical subspecialties have evaluated their own patient populations to determine specific predictors of risk. While these investigations have some degree of interspecialty variability, there are commonalities among them, including age, American Society of Anesthesiologists classification of 3 or greater, congestive heart failure, obstructive sleep apnea, chronic obstructive pulmonary disease, obesity, preoperative anemia, and malnutrition [17–20]. Surgical factors that contribute to risk include surgical site, prolonged surgical duration, and emergency surgery [11, 18, 21].

Numerous scoring systems have been devised which incorporate many of the risk factors described above, including the STOP-BANG questionnaire, to assess the risk for obstructive sleep apnea [22–24], as well as the SPORC (Score for the Prediction of postoperative Respiratory Complications) and ARISCAT (Assess Respiratory Risk in Surgical patients in Catalonia) scores, which were formulated to predict the risk of reintubation and postoperative respiratory complications, respectively [20, 25] (Table 1). While these risk stratification systems have been validated and offer value in assessing patients with comorbidities, each has limitations. The STOP-BANG questionnaire, while useful in identifying and stratifying the severity of obstructive sleep apnea, has not been consistently shown to be a reliable predictor of postoperative hypoxemia [26]. The SPORC focuses on predictors for reintubation but may not be as sensitive in identifying patients with postoperative respiratory compromise who require less invasive intervention. The ARISCAT score, which incorporates more surgical factors, is a population-based score, and its generalizability for use in populations that vary significantly from that of Catalonia, Spain has not been fully assessed [27]. Ultimately, while valuable, these scoring systems do not provide clinicians with a reliable and ongoing method by which to stratify all patients, particularly young and otherwise healthy patients, who may also develop postoperative respiratory impairment. This is likely attributable to the fact that the impact of intra- and postoperative events, medications, and monitoring strategies are not fully incorporated into these risk stratification tools.

## 5. Opioids and the Postoperative Patient

Despite interest in multimodal analgesic therapy, opioids remain the cornerstone of pain management medication strategies. Over the past 2 decades, postoperative opioid use has escalated significantly in response to concerns regarding the undertreatment of pain and the resultant incorporation of pain scales into patient assessments [28]. Studies performed subsequent to the institution of these measures reported an increase in over sedation and opioid-related adverse respiratory events, some with disastrous consequences [29, 30].

These studies, in conjunction with sentinel event reports, spurred the Joint Commission to release Sentinel Event 49, which mandated that hospitals and providers implement policies and procedures to promote safe opioid use and prevent associated adverse events [31]. A recent closed claims analysis of opioid-induced respiratory depression further corroborated these concerns, finding that death and severe brain damage occurred in 55% and 22% of cases, respectively [3]. In addition to excessive opioid dosing, the closed claims investigators found that multimodal opioid administration, continuous infusions, concomitant administration of other sedating medications, and multiple prescribers contributed to these events. Furthermore, 97% of these cases were deemed preventable by adjustments in medication administration or with enhanced monitoring. Interestingly, a recent large database study did not appreciate differences in postoperative hypoxemia when comparing short versus long-acting opioids administered via patient-controlled analgesia systems [32]. This may be attributable to the fact that opioids cause more ventilatory impairment than hypoxemia, and the latter may be a very late manifestation of opioid-induced respiratory depression.

## 6. Postoperative Monitoring Strategies

Given that prediction is an imperfect science, and with opioid therapy remaining the foundation of postoperative pain management, attention has also been directed toward optimization of postoperative monitoring. Multiple studies have demonstrated the inadequacies associated with intermittent assessment of vital signs. In one study, bedside nurses obtained intermittent vital signs while remaining blinded to continuous pulse oximetry monitoring. Notably, 38% of patients experienced hypoxemia (peripheral saturations <90%) for periods of greater than 1 hour, while 27% were hypoxemic for more than 2 hours. In contrast, only 5% of hypoxemic episodes were noted and documented during nursing assessments, which suggests an inadequacy of intermittent monitoring strategies to identify prolonged periods of postoperative hypoxemia [9]. The previously mentioned closed claims analysis found that of the reported adverse respiratory events, respiratory monitoring was not utilized in 53% of cases, nursing assessments were inadequate in 31%, and 42% of respiratory events occurred within 2 hours of a nursing assessment [3]. These investigations highlight the lack of adequate patient monitoring on surgical wards and expose an area of opportunity to improve patient safety.

There is some evidence to suggest that enhanced monitoring may be a method to improve patient safety in the postoperative period. In one study, continuous postoperative monitoring of vital signs was performed coupled with a nursing alert system on an orthopedic surgery floor. Before-and-after analysis suggested that the use of this system was found to decrease both the number of respiratory rescue events and the number of ICU transfers. Investigators found that this enhanced monitoring strategy translated into cost savings by preventing 135 ICU days per year [33].

As a result, multiple agencies, including the Anesthesia Patient Safety Foundation, advocate continuous monitoring for all postoperative patients. However, there is ongoing debate regarding target populations, which vital signs to monitor, and the optimal duration for monitoring. The most obvious parameters to monitor appear to be a combination of oxygen saturation and ventilation. There may be value in incorporating continuous blood pressure monitoring as part of the postoperative monitoring strategy as it is unlikely that respiratory events occur in isolation without hemodynamic consequences. One barrier to implementation of more advanced monitoring systems, beyond the capital investment required, is the development of alarm fatigue, which can result from excessive triggering of alarms due to technical issues, signal degradation, and inadvertent removal by patients. Ideally, appropriate algorithms would be required to filter errant vital signs, accurately identify aberrations, and appropriately alert bedside providers. Additionally, patients may feel encumbered or tethered to monitoring systems for prolonged periods of time, which could impede comfort and impair mobility. Therefore, postoperative monitoring may be optimally utilized during a higher risk time period. While the 4 to 6 hours following PACU dismissal appear to be the highest risk period; the vast majority of adverse respiratory events occur within the first 24 hours following the end of anesthesia [3–5]. Needless to say, this represents a significant area of opportunity for improvement.

## 7. Critical Care Resources and Strain

Critical care resources are often required in the management of patients with postoperative respiratory impairment. Audits of intensive care unit (ICU) admissions suggest that 17–47% of unplanned ICU admissions have a respiratory indication, and that the rate of unplanned ICU admissions is up to 91% higher in patients with postoperative pulmonary complications [34–37]. While some of these admissions may be unavoidable, there are published data to suggest that a significant percentage may be. All ICU admissions have the potential to create excessive demand on the limited supply of critical care resources, termed *ICU capacity strain* [7], but preventable admissions do so unnecessarily. Increasingly, ICUs are operating near capacity, with 90% unable to immediately provide a bed when required, and occupancy rates are projected to increase in the coming years [38–40]. There is ample evidence to suggest that the clinical consequences for patients with respiratory depression may be severe, which could translate into extended period of ICU care. Therefore, capacity strain may not be limited to the day of an unplanned or avoidable admission but could remain a persistent concern for the duration of the patient's stay.

Beyond cost, intensive care unit capacity strain can generate multiple untoward consequences that can adversely impact the care of other patients treated at a given institution (Table 2). Unplanned ICU admissions during times of capacity strain can lead to both unplanned and potentially premature ICU discharges, which has been associated with increased readmissions and increased mortality, for both admitted and prematurely discharged patients [41–44].

Additionally, excessive demand on ICU resources requires an adjustment in triaging paradigms, and bed rationing occurs. With limited capacity, both ICU admissions and discharges tend to be of higher acuity [45]. Up to one-third of ICU admission refusals occur when ICUs are operating at capacity [46], and patients who require critical care services have a lower probability of admission. One study found that when the investigators' ICU operated at >90% capacity, eligible patients had a 9% decrease in the likelihood of admission; when operating at 100% capacity, there was a 47% decrease [47].

ICU capacity strain can also result in a delay in the provision of critical care services, and critically ill patients may need to remain in a lower acuity environment for prolonged periods of time. Both the postoperative and emergency medicine literature have demonstrated that delays, particularly those in excess of 6 hours, are associated with an increase in ICU mortality [48–51] and hospital length of stay [51, 52]. This is likely due to the fact that critically ill patients derive the greatest benefit from ICU management in the early stages of deterioration [53].

Capacity strain in the ICU can also cause perturbations in provider workflow and limit the amount of time that critical care physicians can spend with each patient [54] and in completing other necessary activities, such as documentation [7]. Furthermore, they may be forced to manage critically ill patients in lower acuity locations, particularly in cases of premature ICU discharge. There is also evidence of degradation in quality metrics, such as appropriate stress ulcer prophylaxis [55]. This is corroborated by published literature from emergency medicine, which suggests that capacity strain results in delays in antibiotic administration and less frequent pain assessments [56, 57].

## 8. Conclusion

Postoperative respiratory impairment can have adverse consequences for afflicted patients as well as significant downstream effects that impact care delivery to others. Anesthesiologists, in an expanding role as perioperative physicians, have a multitiered responsibility in ensuring that patients safely transition into the postoperative period. This is particularly important during the highest risk period, which occurs as patients transfer from the PACU to the postoperative ward. Preoperatively, patients should be screened for respiratory vulnerability with the understanding that both intra- and postoperative events must also be considered in risk assessment. Intraoperative management, including medication administration, ventilatory strategies, and volume assessment should be optimized based on patients' comorbidities. Given limitations in risk prediction scores, appropriate vigilance and monitoring strategies should be employed, particularly during high-risk time periods, and anesthesiologists should take an active role in determining the optimal monitoring and acuity locations for patients upon PACU discharge.

The practice of anesthesiology now encompasses the entirety of perioperative medicine, with over half of anesthesia practices providing critical care services at their respective institutions [58]. Anesthesiologists have a unique opportunity to leverage specialty expertise in physiology, pharmacology, and pain management and a close relationship with surgical colleagues to promote patient safety in the perioperative period.

## Figures and Tables

**Table 1:** Respiratory risk stratification scoring systems [20, 22, 25].

STOP-BANG Questionnaire [22]	*Use*	Screen/stratify risk of OSA	
	*Components*	Snoring	BMI > 35 kg/m^2^
		Tiredness	Age > 50 years
		Observed apnea	Neck circumference (large size)
		High blood pressure	Gender (male)
	*Interpretation*	0–2: low risk	
		3-4: intermediate risk	
		5–8: high risk	

SPORC (Score for the Prediction of Postoperative Respiratory Complications [25])	*Use*	Risk stratification for development of postextubation respiratory failure requiring reintubation	
	*Components*	ASA score ≥ 3	3 points
		Emergency procedure	3 points
		High-risk service	2 points
		Congestive heart failure	2 points
		Chronic pulmonary disease	1 point
	*Interpretation*	0 points	Reintubation probability 0.1%
		3 points	Reintubation probability 0.5%
		5 points	Reintubation probability 1.5%
		7 points	Reintubation probability 4.2%
		9 points	Reintubation probability 11.2%

ARISCAT (Assess Respiratory Risk in Surgical Patients in Catalonia [20])	*Use*	Risk stratification for the development of postoperative pulmonary complications	
	*Components*	Age	
		≤50 years	0 points
		51–80 years	3 points
		>80 years	16 points
		Preoperative oxygen saturation	
		≥96%	0 points
		91–95%	8 points
		≤90%	24 points
		Other clinical risk factors	
		Respiratory infection (in prior month)	17 points
		Preoperative hemoglobin ≤10 g/dL	11 points
		Emergency surgery	8 points
		Surgical incision	
		Upper abdominal	15 points
		Intrathoracic	24 points
		Duration of surgery	
		<2 hours	0 points
		2–3 hours	16 points
		>3 hours	23 points
	*Interpretation*	<26 points: low risk	
		26–44 points: moderate risk	
		≥45 points: high risk	

**Table 2:** Consequences of ICU capacity strain.

Higher acuity admissions
Higher acuity discharges
Premature/unplanned ICU discharges
Increase in ICU admission refusals
Delay in ICU admission
Mismatch in patient acuity and treatment location
Increase in mortality
Increase in cost
Disruption of provider workflow
Decreased time spent caring for each patient
Degradation in patient care
